# The role of cytostatic in oxidative stress reactions

**DOI:** 10.3389/fonc.2025.1667522

**Published:** 2025-10-13

**Authors:** Renata Polaniak, Aleksander Kwiatkowski, Michał Górski, Elżbieta Grochowska-Niedworok, Małgorzata Latocha

**Affiliations:** ^1^ Department of Dietetics, Division of Human Nutrition Faculty of Public Health in Bytom Medical University of Silesia in Katowice, Bytom, Poland; ^2^ Department of Human Anatomy, Faculty of Medical Sciences in Katowice, Medical University of Silesia in Katowice, Katowice, Poland; ^3^ Department of Chronic Diseases and Civilization-Related Hazards, Faculty of Public Health in Bytom, Medical University of Silesia in Katowice, Katowice, Poland; ^4^ Health Public School in Nysa, Nysa, Poland; ^5^ Department of Cell Biology, Faculty of Pharmacy with Division of Laboratory Medicine in Sosnowiec, Medical University of Silesia in Katowice, Sosnowiec, Poland

**Keywords:** cytostatic, cisplatin, doxorubicin, reactive oxygen species (ROS), cancer

## Abstract

Cytostatic drugs are widely applied in cancer therapy. Among the most commonly used agents are anthracyclines, such as doxorubicin, and platinum (II) complexes, including cisplatin, carboplatin, and oxaliplatin. Treatment with cytostatic drugs has been shown to enhance the mitochondrial production of reactive oxygen species (ROS). Cells regulate redox homeostasis through scavenging systems, with antioxidant enzymes playing a crucial role in neutralizing ROS. Key enzymes involved in this defense include superoxide dismutase, catalase, and glutathione S-transferase, whose activity may be modulated under oxidative stress conditions. Previous research has documented the effects of cytostatic drugs on cancer cell cultures *in vitro*, as well as the corresponding alterations in antioxidant enzyme activity observed under these conditions.

## Introduction

In healthy cells, oxidative stress is tightly regulated by compartmentalized antioxidant systems-glutathione, thioredoxin/peroxiredoxin cycles, catalase in peroxisomes, and SOD isoforms in cytosol, mitochondria, and extracellular space -maintaining ROS at low, signaling-competent levels (1, 2). In cancer cells, oncogenic signaling (e.g., MYC, RAS), mitochondrial dysfunction, rapid proliferation, and hypoxia/reoxygenation events cumulatively elevate ROS. This persistent redox shift fuels genomic instability and malignant progression, yet also creates therapeutic liabilities by lowering the threshold for ROS-mediated cell death (1).

Key differences include: (a) increased basal mitochondrial ROS and NADPH oxidase activity; (b) altered redox buffering (high glutathione turnover, reliance on pentose phosphate pathway for NADPH); (c) adaptive upregulation of antioxidant enzymes (e.g., MnSOD, GPx) in drug-resistant phenotypes; and (d) microenvironmental factors (hypoxia, inflammatory cytokines, metal ions) that modulate oxidative fluxes. These distinctions are central to interpreting the effects of cytostatics that further perturb redox homeostasis (2, 3).

This review synthesizes enzymatic and non-enzymatic antioxidant responses to major cytostatics and explicitly contrasts outcomes in normal versus cancer cells. We integrate scattered *in vitro* findings, highlight translational gaps, and outline therapeutic opportunities and pitfalls in targeting antioxidant networks.

**Figure 1:**
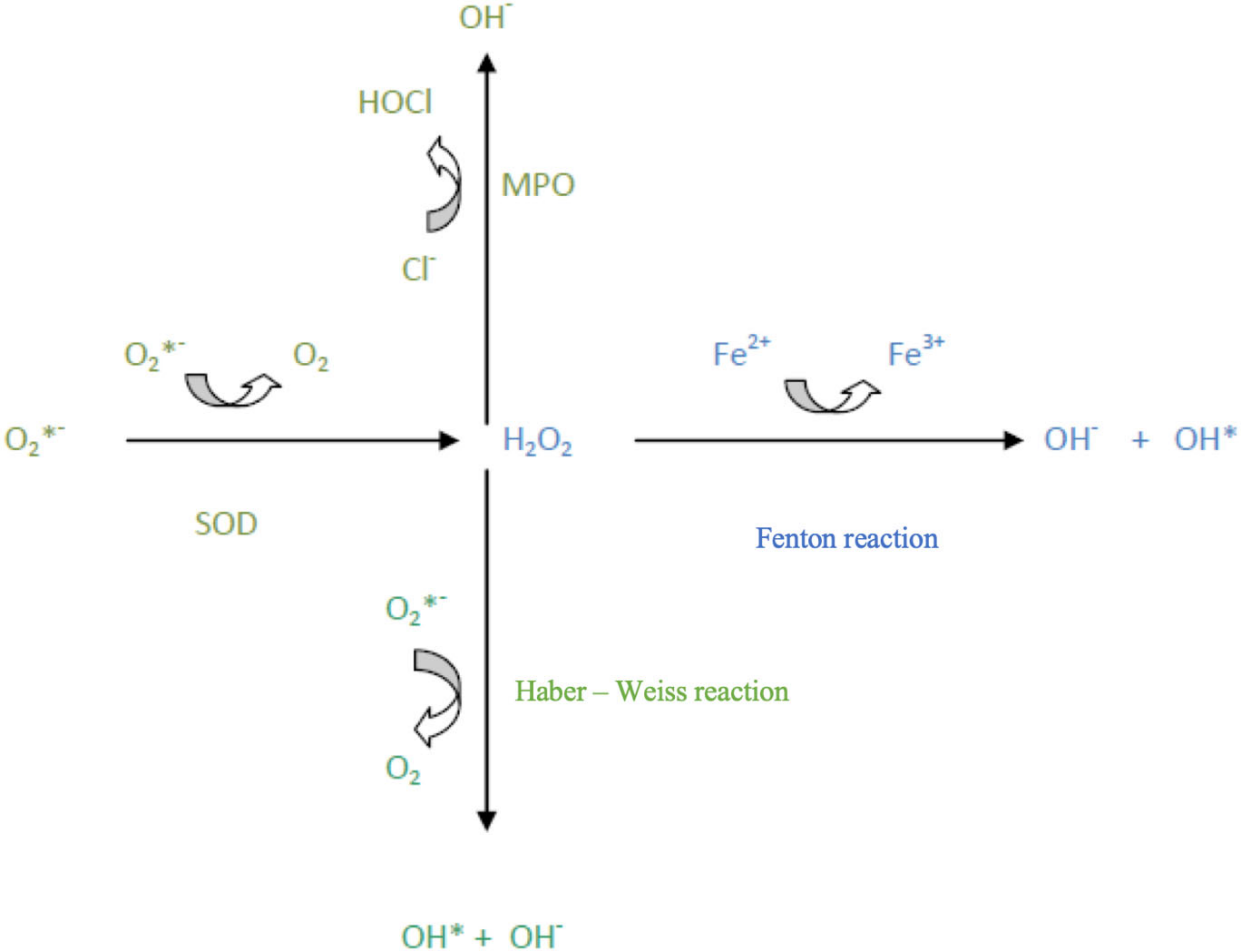
Reactive oxygen species (ROS) transformations.

Oxidative stress arises within a cell due to an imbalance between the formation of free radicals (FR) – specifically, reactive oxygen species (ROS) – and the cell’s ability to eliminate them and repair the damage they cause. Reactive oxygen species interact with nucleic acids, causing DNA mutations, which disrupt replication and transcription. Consequently, repair mechanisms are activated, or cellular death occurs. Additionally, the accumulation of mutations in genetic material often initiates carcinogenesis through uncontrolled proliferation of cells that are unresponsive to pro-apoptotic signals (1, 2, 4) (**Figure 1**).

A well-known free radical process is lipid peroxidation, which involves the oxidation of polyunsaturated fatty acids. This may affect phospholipids of cell, nuclear, mitochondrial, and peroxisomal membranes (2). Lipid peroxides form during this process, participating in further lipid peroxidation, with the final reaction products being phospholipid dimers and aldehydes. An example of a lipid peroxidation marker is malondialdehyde (MDA) (2). Free radicals also react with proteins, often resulting in altered or lost enzymatic functions (5). Thus, free radicals damage specific cellular structures and may lead to cell death. Through their action, they may also affect cell proliferation, differentiation, or interfere with signal transduction (2, 6).

In cells, free radicals primarily arise from oxygen-dependent processes in mitochondria, producing reactive oxygen species (ROS) such as the superoxide anion (O_2_-), hydroxyl radical (·OH), and hydroperoxide radical (HO_2_.) (3). This necessitates cellular defense mechanisms to counteract free radical attacks and repair induced damage (4). The cell has multiple defense mechanisms, both non-enzymatic, involving antioxidants such as vitamins C, E, and A, and enzymatic, involving antioxidant enzymes (2, 7, 8).

## The role of cytostatics in oxidative stress reactions

### Antioxidant enzymes

Key antioxidant enzymes in the cell include superoxide dismutase (SOD) [EC 1.15.1.1]; the zinc-copper isoenzyme of superoxide dismutase, SOD1 (Cu/ZnSOD), which is primarily found in the cytoplasm of liver, testes, kidney, nervous system, erythrocytes and manganese-dependent superoxide dismutase, SOD2 (MnSOD), located in the mitochondrial matrix, peroxisomes, and to a lesser extent, extracellularly. The third SOD isoenzyme is extracellular superoxide dismutase, SOD3 (EC-SOD), predominantly found in the extracellular space, with activity observed in blood, lymph, interstitial fluid, and cerebrospinal fluid (9).

These enzymes are metalloenzymes, containing a metal ion in their active center that alternately reduces or oxidizes, catalyzing the two-step dismutation of the superoxide anion, a highly active free radical, into oxygen and hydrogen peroxide (9). To illustrate the critical role of manganese-dependent superoxide dismutase, we can refer to research by Zhang et al. (10), who examined biochemical transformations in SOD2-/- mouse fibroblasts, cells lacking this enzyme. *In vivo*, such a phenotype is lethal, and researchers found that the absence of mitochondrial SOD activity disrupts cellular signaling pathways, slowing growth and proliferation (10) (**Table 1**).

**Table 1:** Comparative roles of key antioxidant enzymes in healthy vs. cancer cells (1–13).

Enzyme	Role in Healthy Cells	Role in Cancer Cells
Superoxide Dismutase (SOD)	Neutralizes superoxide anions to hydrogen peroxide	Overexpressed in some cancers, linked with drug resistance
Catalase (CAT)	Breaks down hydrogen peroxide into water and oxygen	Altered activity contributes to oxidative imbalance
Glutathione Peroxidase (GPx)	Reduces hydrogen peroxide and lipid peroxides	Elevated in resistant cells, enhances survival
Enzyme	Role in Healthy Cells	Role in Cancer Cells
Superoxide Dismutase (SOD)	Converts O2•– to H2O2; maintains signaling-level ROS	Often upregulated; may correlate with chemoresistance (MnSOD, SOD1)
Catalase (CAT)	Detoxifies H2O2 in peroxisomes	Lower/redistributed in some tumors; imbalance favors H2O2 signaling
Glutathione Peroxidase (GPx)	Reduces H2O2 and lipid peroxides using GSH	Elevated in resistant lines; supports survival under cytostatic stress
Glutathione Reductase (GR)	Recycles GSSG to GSH (NADPH-dependent)	Lower activity reported with some drugs; redox bottlenecks appear
Glutathione S-Transferase (GST)	Conjugates electrophiles with GSH for detoxification	GSTP1 overexpression linked with proliferation and drug resistance

Another antioxidant enzyme is catalase (CAT) [EC 1.11.1.6], which catalyses the dismutation of hydrogen peroxide into water and oxygen. At high hydrogen peroxide concentrations, catalase primarily exhibits catalase activity; at low concentrations H_2_O_2_, it displays peroxidase activity (9).

It is possible to encapsulate superoxide dismutase or catalase molecules in large unilamellar liposomes of about 110 nm in size. These resulting enzymosomes are used to deliver antioxidant enzymes directly to target cells, increasing their biodistribution. Studies in rats have shown that enzymosomes reduce oxidative stress induced by radiotherapy and positively impact retinal cells in new-born rats (11).

The third group of antioxidant enzymes, selenoperoxidases, plays a significant role in defense mechanisms against free radicals and contains selenocysteine in the enzyme’s active center. The main representative of this group is glutathione peroxidase (GPx) [EC 1.11.1.9], which reduces hydrogen peroxide and organic peroxides. It exists in several isoforms: cytosolic (cGPx), gastrointestinal (giGPx), plasmatic (pGPx), nuclear (spGPx), and as phospholipid hydroperoxide peroxidase (phGPx) (9). A recently described glutathione peroxidase (snGPx) protects sperm DNA from oxidative damage and is involved in chromatin condensation (9). Glutathione peroxidase activity depends on cellular glutathione, which is consumed in the reaction and can be regenerated by glutathione reductase (GR) [EC 1.6.4.2] with NADPH+H^+^ (9).

Another enzyme in this group, glutathione S-transferase (GST) [EC 2.5.1.18], catalyzes the conjugation of electrophilic compounds with glutathione, including harmful metabolites like bilirubin, fatty acid peroxides, and xenobiotics such as cytostatic drugs. Conjugation makes these compounds water-soluble, allowing safe excretion via urine (9). GST has multiple isoforms, with GSTP1 receiving significant attention in literature due to its overexpression in various cancer cell types (12). Researches (12, 13) demonstrated that elevated GSTP1 activity in human HCT 116 colon cancer cells contributes to their uncontrolled proliferation.

Reactive oxygen species (ROS) are continuously generated in cells, accompanied by antioxidant processes that maintain a balance. Oxidative stress, a disruption of this balance, represents the cell’s response to physical, chemical, and biological factors (2, 7). Studies have shown that irritants, such as asbestos (14) or tobacco smoke (15), increase free radical production within cells. This also occurs in response to certain drugs. This article examines changes in antioxidant enzyme activity under the influence of cytostatic drugs on cells (16).

### Therapeutic applications and challenges in targeting antioxidant enzymes

Targeting antioxidant enzymes (e.g., SOD2, GPx, GST) can sensitize tumors to cytostatics by pushing ROS beyond cytotoxic thresholds. Examples include GST inhibitors that prevent drug conjugation and efflux, or modulation of GSH synthesis to transiently lower cellular buffering capacity (17).

Certain challenges associated with this have also been identified: (a) therapeutic window—systemic suppression of antioxidants risks normal-tissue toxicity (cardiotoxicity, neurotoxicity); (b) compensatory rewiring—cancer cells upregulate parallel redox pathways; (c) pharmacokinetics—achieving tumor-selective delivery; (d) biomarker selection—lack of standardized, clinically actionable ROS/redox biomarkers to guide patient selection (17, 18).

Despite these limitations, it is also worth pointing out the significant opportunities: (a) liposomal and pegylated formulations (e.g., PLD) and enzymosome carriers may co-deliver cytostatics and redox modulators; (b) radiochemotherapy regimens can exploit ROS bursts; and (c) adaptive dosing based on early redox readouts (e.g., MDA, 4-HNE adducts) may optimize efficacy while limiting harm (17–19).

### Cytostatic drugs

Cytostatics are a chemically diverse group of drugs with anticancer activity. Chemotherapy regimens are based on multi-center clinical trials (20, 21). Types of chemotherapy include induction chemotherapy to reduce tumor mass before planned surgery, postoperative adjuvant chemotherapy, and palliative chemotherapy for inoperable cancers (22). Numerous studies have investigated the impact of cytostatics on cellular mechanisms and their induction of cell death (23).

Among clinically significant cytostatic groups, this work focuses on cisplatin and its derivatives, carboplatin and oxaliplatin.

**Figure 2:**
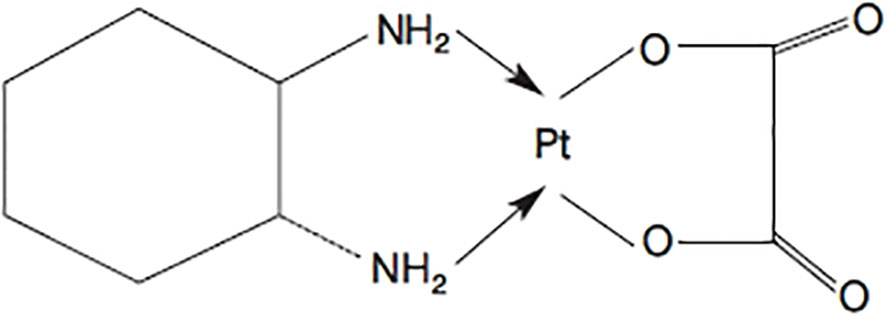
Ryc. I. Cisplatyna (cis-diaminodichloroplatyna).

*Cisplatin (CIS*) is a fundamental chemotherapeutic used to treat various stages of testicular and ovarian cancers, as well as bladder, esophageal, advanced head and neck cancers, and both small-cell and non-small-cell lung cancer. It is a Platinum (II) compound with two chloride ligands and two NH_3_ residues in a *cis* configuration, acting by alkylating DNA and forming intra- and inter-strand bonds, thus inhibiting DNA replication and transcription to RNA (24, 25). Notably, the trans-isomer of this compound lacks anti-cancer activity, though certain trans-platinum derivatives exhibit anti-carcinogenic properties (26) (**Figure 2**).

With the increasing resistance of cancer cells to cisplatin, it is necessary to search for its new derivatives (16). Among the various platinum compounds, carboplatin and oxaliplatin are mainly used in treatment. Like cisplatin, these are alkylating compounds but exhibit different pharmacokinetics and tend to cause fewer side effects (27). Both maintain the *cis* configuration in their structure, differing in substituents (24).

Carboplatin contains two NH_3_ ligands in a cis conformation and a cyclobutane-1,1-dicarboxylic acid residue. This drug is used in treating ovarian cancer, cervical cancer, testicular cancer, small-cell and non-small-cell lung cancer, as well as squamous cell carcinoma of the head and neck (24, 25, 28). There are numerous reports of cross-resistance of cancer cells to cisplatin and carboplatin (25).

Oxaliplatin is distinguished in its structure by 1,2-diaminocyclohexane and an oxalate group (24). It is a cisplatin derivative used to treat rectal and colon cancer at various stages, typically combined with 5-fluorouracil and folic acid in therapeutic regimens (25, 29). It is also present in chemoradiotherapy protocols for rectal cancer (30). The cytotoxic properties of oxaliplatin against various cancer types, including those resistant to cisplatin, have been frequently described in the scientific literature (25).

Another group of cytotoxic drugs includes anthracycline antibiotics. These are used in cancer treatment due to their cytotoxic properties. Their mechanism involves preventing DNA transcription by embedding into its helix and inhibiting the action of topoisomerase II (24, 31).

**Figure 3:**
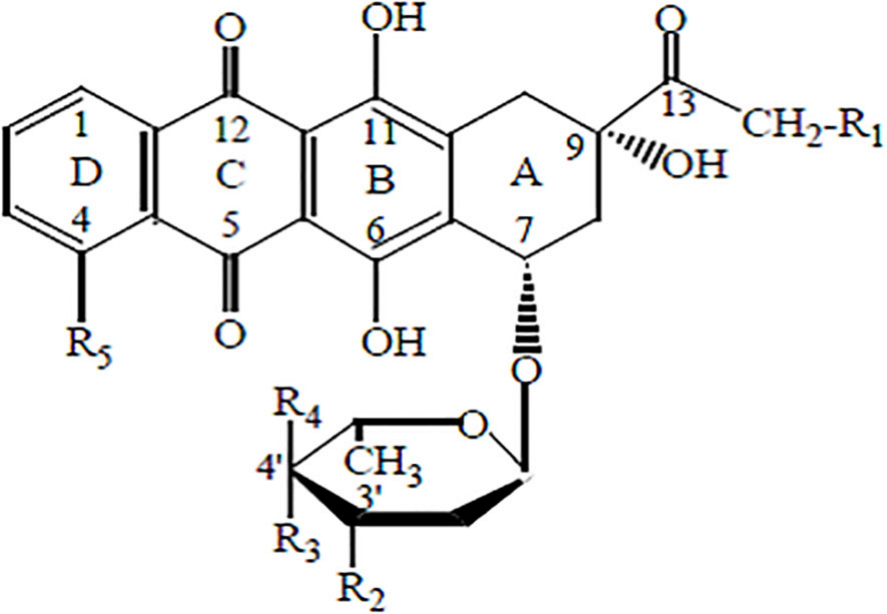
Ryc. II. Chemical structure of anthracyclines.

The main representative of anthracycline antibiotics is doxorubicin (DOX), also known as adriamycin (**Figure 3**).

Its molecule consists of a four-ring aglycone structure and a sugar moiety. The substituents include hydroxyl, carbonyl, and amino groups. This antibiotic is widely used in chemotherapy for cancers of the breast, ovary, endometrium, thyroid, bladder, stomach, prostate, liver, as well as leukemias, immunological malignancies, pediatric cancers such as Wilms’ tumor and neuroblastoma, and AIDS-related cancers, including Kaposi’s sarcoma (32, 33). The drug is typically administered intravenously as part of the so-called “red chemotherapy.” Intravesical administration of doxorubicin is also possible for the treatment of bladder cancer (34).

Numerous studies highlight the significant cardiotoxicity of doxorubicin and the frequent development of cellular resistance (35–37). As a result, its liposomal formulation—liposomal doxorubicin—is increasingly used in therapy, proving more advantageous for treating patients with cancer recurrence (31). Additionally, pegylated liposomal doxorubicin (PLD) is available and utilized in the treatment of metastatic breast cancer, ovarian cancer, and highly vascularized tumors such as Kaposi’s sarcoma. This modification extends the drug’s circulation time and enhances its bioavailability (28, 32, 38). Doxorubicin is used both in monotherapy and in combination with other cytostatic agents, such as cyclophosphamide (39). Currently, numerous doxorubicin analogs with varying toxicity profiles and pharmacokinetics are employed in treatment (24).

The literature contains extensive research on the effects of cytostatic drugs on cancer cells in *in vitro* cultures. Studies on the mechanisms of doxorubicin action on cancer cells have been conducted using human lung adenocarcinoma cell line A549 (40), as well as human ovarian teratoma cell line PA-1 and ovarian cancer cell line CA5171 (41), and human breast adenocarcinoma cell line MCF-7 (36). *In vitro* investigations have also explored the metabolism of human colorectal cancer cell line HCT-116, T-cell acute lymphoblastic leukemia cell line Jurkat, and human promyelocytic leukemia cell line HL-60 under the influence of doxorubicin and cisplatin (42). Georgakis et al. (43) examined the apoptosis mechanisms in Hodgkin lymphoma cell lines HD-MyZ, HD-LM-2, L-428, and KM-H2 in response to doxorubicin. Kachadourian et al. (42) studied the effects of cisplatin on human lung adenocarcinoma cell line A549 in *in vitro* culture (42). Similarly, studies on the effects of carboplatin on cancer cells in *in vitro* cultures were conducted on human melanoma cell line Me45 (44) and human glioblastoma cell line SNB19 (45) (**Table 2**).

**Table 2:** Selected studies on cytostatics and oxidative stress (models, exposures, findings) (10, 44–50).

Study	Model	Drug/Exposure	Key finding (oxidative/enzymatic)	Model type
Bułdak et al. (44)	Me45 melanoma cells	Carboplatin (10–100 µg/mL)	↑ GPx; ↓ GR; oxidative adaptation	*in vitro*
Bułdak et al. (46)	Me45 melanoma cells	Etoposide (20–200 µg/mL)	↑ MnSOD, CuZnSOD, CAT, GPx; ↓ GR	*in vitro*
Polaniak et al. (47)	AT478 squamous carcinoma	Holoxan (10–40 µg/mL)	↑ MnSOD and CuZnSOD	*in vitro*
Polaniak et al. (45)	SNB19 glioblastoma	Carboplatin (two doses)	↑ Total SOD, MnSOD, GPx (72 h > 24 h)	*in vitro*
Alexandre et al. (48)	CT26 colorectal cells	Oxaliplatin + mangafodipir	SOD-mimic ↑ chemo cytotoxicity; ↓ hematotoxicity	*in vitro*
Dusre et al. (49)	MCF-7 breast cancer	Mitomycin C & analogues	Cross-links/FR formation; links to resistance	*in vitro*
Samuels et al. (50)	Human sarcoma lines	Doxorubicin	↑ GPx in resistant line (×6)	*in vitro*
Zhang et al. (10)	Mouse fibroblasts (SOD2−/−)	Genetic deletion	Loss of MnSOD disrupts signaling; growth defects	*in vitro*/lethal *in vivo*

↑, increase; ↓, decrease.

In the presence of cytostatic drugs, including platinum derivatives (51) and doxorubicin (33), the levels of reactive oxygen species (ROS) increase within cells (29). Depending on the cell type, drug concentration, or experimental conditions, the cellular response to oxidative stress varies (29, 51).

Cancer cells are characterized by an inherently higher baseline level of reactive oxygen species, making them more susceptible to oxidative stress (15, 52). By studying changes in the activity of superoxide dismutase isoenzymes, catalase, and glutathione peroxidase in cells cultured *in vitro* under the influence of a cytostatic drug, the extent of oxidative stress induced by the drug can be measured. Numerous reports in the literature indicate that an increase in antioxidant enzyme activity contributes to greater cellular resistance to cytostatic drugs. L’Ecuyer et al. (53) examined the effects of anthracycline antibiotics on rat cardiomyocytes of the H9C2 cell line, suggesting that selective overexpression of antioxidant enzymes in cardiomyocytes could reduce the cardiotoxicity of chemotherapy (53).

Zhong et al. (54) investigated the effects of antioxidants *in vitro* on human prostate adenocarcinoma RWPE-2 cells, depending on the expression of manganese superoxide dismutase (MnSOD). Their study demonstrated that cells overexpressing MnSOD exhibited greater sensitivity to the cytotoxic effects of buthionine sulfoximine, a compound that reduces intracellular glutathione levels, and vitamin C. Conversely, these cells showed reduced sensitivity to selenium compounds. The same effect was observed when the cells were treated with an exogenous MnSOD analog. Considering that glutathione peroxidase is a selenium-dependent enzyme, the authors concluded that MnSOD overexpression exerts both pro-oxidative and antioxidative effects, depending on the activity of other antioxidant enzymes. This finding underscores the need for further research into the activity of antioxidant enzymes in cancer cells and their interrelations, as this may have implications for the outcomes of anticancer therapies (54).

The literature contains numerous reports of higher antioxidant enzyme activity, such as GPx and MnSOD, in cancer cells resistant to cytostatic drugs (49, 50, 55, 56). As early as the 1990s, Dusre et al. (49) demonstrated that *in vitro* doxorubicin-resistant MCF-7 breast cancer cells exhibited higher glutathione peroxidase activity compared to cells that were not resistant to the cytostatic. Similarly, Samuels et al. (50), investigating GPx activity in two human sarcoma cell lines with differing resistance to doxorubicin, found that GPx activity was six times higher in the cell line less sensitive to apoptosis induced by doxorubicin treatment. This highlights the role of antioxidant enzymes in the development of drug resistance in cancer cells (49, 50).

Bułdak et al. (44), investigating the effects of carboplatin on antioxidant activity in malignant melanoma Me45 cells, demonstrated an increase in glutathione peroxidase (GSH-Px) activity following carboplatin treatment. Specifically, GSH-Px activity increased to 125.2 ± 12.1 IU/L and 99.1 ± 13.3 IU/L after 24 hours of exposure to carboplatin at concentrations of 10 µg/mL and 100 µg/mL, respectively, compared to 91.6 ± 12.1 IU/L in the control group. Additionally, a reduction in glutathione reductase (GR) activity was observed, with GR activity decreasing to 8.49 ± 0.31 IU/L after 24 hours of treatment with carboplatin at a concentration of 10 µg/mL, compared to 9.49 ± 0.49 IU/L in the control group.

In a related study by Bułdak et al. (46), examining the increase in antioxidant enzyme activity in malignant melanoma Me45 cells cultured *in vitro* following treatment with etoposide—a cytotoxic drug from the podophyllotoxin derivatives group—a significant increase in the activity of MnSOD, CuZnSOD, CAT, and GSH-Px was observed after 24 hours of exposure to etoposide at concentrations of 20 µg/mL and 200 µg/mL, compared to the control group. Simultaneously, GR activity decreased in both experimental groups. These results suggest an adaptive response of the cells to oxidative stress induced by the cytostatics.

There are scientific reports on the effects of other cytostatic drugs on antioxidant enzyme activity in cells. In the study by Polaniak et al. (47), an increase in MnSOD isoenzyme activity was observed in AT478 squamous carcinoma cells treated with holoxan at concentrations of 10 µg/mL and 40 µg/mL for 24 hours, compared to the control group, with increases of 9.2 NU/mL and 14.69 NU/mL, respectively, versus 1.2 NU/mL in the control group. Additionally, an increase in Cu/ZnSOD isoenzyme activity was noted in these cells, with levels of 3.7 NU/mL and 4.1 NU/mL versus 1.4 NU/mL in the control group.

In another study on changes in the activity of the pro-oxidant/antioxidant enzyme system in human glioblastoma SNB19 cells under the influence of carboplatin, Polaniak et al. (45) found that the total SOD activity in cells treated with carboplatin at two different concentrations was higher compared to controls at both 24 and 72 hours. The highest activity of SOD, MnSOD, and GSH-Px was observed in samples treated with the higher concentration of carboplatin after 72 hours. The activity of the SOD2 isoenzyme and GSH-Px was higher in all experimental groups at 72 hours compared to 24 hours. In contrast, the activity of the copper-zinc superoxide dismutase isoenzyme (Cu/ZnSOD) in samples exposed to carboplatin at both concentrations was higher than in the control at both 24 and 72 hours. However, in the lower-concentration group, SOD1 activity was lowest at 72 hours (45).

Alexandre et al. (48) investigated the effects of mangafodipir, a substance used as a contrast in magnetic resonance imaging, which acts as a superoxide dismutase (SOD) mimetic and exhibits catalase and glutathione reductase activity, on the cytotoxicity of anticancer drugs. They demonstrated that the use of this oxidative stress modulator increased the cytotoxicity of oxaliplatin and paclitaxel in CT26 colorectal cancer cells in *in vitro* cultures while reducing hematotoxicity. They hypothesized that this effect was due to the antioxidant activity of mangafodipir.

## Current challenges in research

One of the main difficulties is the dual nature of ROS, which can both induce carcinogenesis and promote apoptosis depending on context. Another challenge is tumor heterogeneity: ROS levels and enzyme activities vary significantly across cancer types, complicating universal strategies. Moreover, translating promising *in vitro* findings to *in vivo* and clinical settings remains problematic (57).

Methodological caveats include assay selection (e.g., DCF-DA vs. mitochondria-specific probes), artifact-prone measurements, and endpoint timing (24 h vs. 72 h) that can invert interpretations. Standardized protocols and reference controls are needed to improve reproducibility across labs (58).

Clinical translation hurdles comprise patient heterogeneity, prior therapy exposure reshaping tumor redox landscapes, and difficulty in serially sampling tumors. Liquid biomarkers (lipid peroxidation products, oxidized nucleotides) and imaging surrogates may partially bridge this gap (57–60).

Numerous studies on the mechanisms of action of cytotoxic drugs and their efficacy have focused on exploring the mechanisms of cell resistance to these agents. For years, we have wanted to understand the underlying causes of cancer, its origins, and methods of prevention. In the literature, there is significant interest in studying changes in antioxidant enzyme activity in cancer cell lines during *in vitro* cultures under the influence of cytostatic drugs.

## Future perspectives

Future work should integrate nanotechnology, gene therapy, and ROS-modulating strategies with conventional cytostatics. Personalized medicine approaches based on tumor-specific ROS profiles could improve therapeutic selectivity. Combination therapies, integrating cytostatics with antioxidants or pro-oxidants, may offer synergistic benefits and reduce side effects.

Precision redox oncology will likely rely on composite biomarkers (enzyme activities, GSH/GSSG ratio, redox-sensitive transcriptional signatures) to stratify patients. Adaptive trials can test cytostatics ± redox modulators with early stopping based on toxicity/efficacy readouts.

Engineering advances (stimuli-responsive nanoparticles releasing payloads in high-ROS niches, tumor-penetrating peptides, and mitochondrial-targeted carriers) could widen the therapeutic window, enhancing tumor selectivity while sparing normal tissues.

Integration with immunotherapy is also promising. ROS can remodel antigen presentation and the tumor microenvironment. Rational scheduling may synergize redox modulation with checkpoint blockade or adoptive cell therapies.

## Conclusion

Research on oxidative stress enzyme activity changes provides essential insights into the mechanisms of cytostatic action in cancer cells. A deeper understanding of ROS-antioxidant interactions may enable the design of selective therapies with reduced toxicity. With advancements in gene therapy, targeted drug delivery, and nanomedicine, chemotherapy effectiveness could be significantly improved. Translating these findings into clinical applications will be key to overcoming resistance and improving patient survival.
